# Considerations and recommendations for collaborative research networks in epidemiology: Lessons learned from the diabetes LEAD Network

**DOI:** 10.1017/cts.2024.670

**Published:** 2024-12-16

**Authors:** Tara P. McAlexander, Nora L. Lee, Gina S. Lovasi, Annemarie G. Hirsch, Melissa N. Poulsen, Brian Elbel, Lorna E. Thorpe, D. Leann Long, Leslie A. McClure

**Affiliations:** 1 Department of Epidemiology and Biostatistics, Drexel University Dornsife School of Public Health, Philadelphia, PA, USA; 2 Department of Population Health Sciences, Geisinger, Danville, PA, USA; 3 Department of Population Health, New York University Grossman School of Medicine, New York, NY, USA; 4 Department of Biostatistics and Data Science, Wake Forest University School of Medicine, Winston-Salem, NC, USA; 5 Department of Biostatistics, The University of Alabama at Birmingham School of Public Health, Birmingham, AL, USA; 6 Saint Louis University School of Public Health, St Louis, MO, USA

**Keywords:** Collaborative research, team science, epidemiology, harmonization, research design and methods

## Abstract

Multi-site and multi-organizational teams are increasingly common in epidemiologic research; however, there is a lack of standards or best practices for achieving success in collaborative research networks in epidemiology. We summarize our experiences and lessons learned from the Diabetes Location, Environmental Attributes, and Disparities (LEAD) Network, a collaborative agreement between the Centers for Disease Control and Prevention and research teams at Drexel University, New York University, Johns Hopkins University and Geisinger, and the University of Alabama at Birmingham. We present a roadmap for success in collaborative epidemiologic research, with recommendations focused on the following areas to maximize efficiency and success in collaborative research agreements: 1) operational and administrative considerations; 2) data access and sharing of sensitive data; 3) aligning network research aims; 4) harmonization of methods and measures; and 5) dissemination of findings. Future collaborations can be informed by our experiences and ultimately dedicate more resources to achieving scientific aims and efficiently disseminating scientific work products.

## Introduction

Research is often a collaborative effort. Collaborative teams usually form based on prior joint work among researchers, with complementary expertise from multiple disciplines around a common topic. Some teams, however, are formed by an external entity, such as a funding agency, joining together multiple research teams that may have not previously worked together (usually at different institutions and geographic locations) into a grant-funded network around a central research theme. We herein refer to networks as collaborative research efforts spanning multiple institutions, including research sites and a coordinating center, with a specific charge for achieving unified scientific aims. An advantage of such multisite teams is that each site brings its own well-established research program with potentially varying approaches to the same public health problem. These multisite teams include diverse resources and experiences. Examples of such teams in population health include the Centers for Disease Control and Prevention (CDC)-funded Natural Experiments for Translation in Diabetes study and the National Cancer Institute-funded consortia for Coordinated Collaborative Science [1,2]. While there are many benefits to forming a multisite collaborative team, there are also challenges. Large externally formed collaborations require additional time for communications and coordination of work; teams from different disciplines may initially lack a common vocabulary; and misalignment of perceived goals can generate conflict [3,4]

Over the last half century, scientific teams have expanded in size, as reflected in the growth of author lists on publications [4]. Researchers participating in such networks and network funders can thus benefit from understanding best practices for organizing and operating a collaborative research network in ways that optimize opportunities for scientific innovation. There have been prior efforts to gain insights from multi-institutional scientific research teams, including for health-related studies [5–7]. However, these studies focus on multi-institutional teams that were formed prior to applying for funding to support their efforts. Here, we expand upon this literature to focus on best practices for multisite research collaborations particularly for those that have not worked together previously, with a focus on epidemiological analyses across multiple study populations. We reflect on a multisite collaborative research effort, the Diabetes Location, Environmental Attributes, and Disparities (LEAD) Network.

LEAD was a CDC-funded collaboration of the CDC, Drexel University, New York University Grossman School of Medicine (NYU), the University of Alabama at Birmingham (UAB), and Johns Hopkins University/Geisinger (JHU/G). Drexel University served as the coordinating center, and NYU, UAB, and JHU/G served as study sites. Each site and the Coordinating Center independently responded to a funding opportunity and proposed site-specific research aims to identify modifiable environmental risk factors for new onset type 2 diabetes (T2D). Thus, the funding sponsor defined the scope of the research network. Each institution identified teams and data resources during the development of independent proposals reflective of specific expertise. For example, the NYU site had extensive experience conducting epidemiologic studies using data from the US Department of Veterans Affairs electronic health records (EHRs); the JHU/G site had similar experience using EHRs from Geisinger, a health system in Pennsylvania. In contrast, the team at UAB leveraged expertise and data from two sources: the REasons for Geographic and Racial Differences in Stroke (REGARDS) study, a longitudinal epidemiologic cohort study, and administrative claims from a 5% sample of Medicare beneficiaries. Each site, along with the Drexel Coordinating Center, was funded to form a collaborative research network that leveraged each teams’ expertise and data assets. CDC served as an active research partner, participating in all LEAD Network meetings and contributing authorship on many network-related publications. We iteratively developed and addressed network-wide scientific aims and hypotheses to identify modifiable risk factors driving geographic disparities in new-onset T2D [8].

In this paper, we reflect on our experiences and offer suggestions for using scientific goals, frameworks, and available resources of a multi-site collaborative network to develop a roadmap for success. We summarize barriers to progress and strategies we successfully employed to overcome them related to five key operational and scientific functions of the LEAD Network: 1) operational and administrative considerations; 2) data access and sharing of sensitive data; 3) aligning network research aims; 4) harmonization of methods and measures; and 5) dissemination of findings. These recommendations are especially relevant to process-dependent collaborations that form to address specific scientific questions.

### Operational and administrative considerations

**Figure 1.**
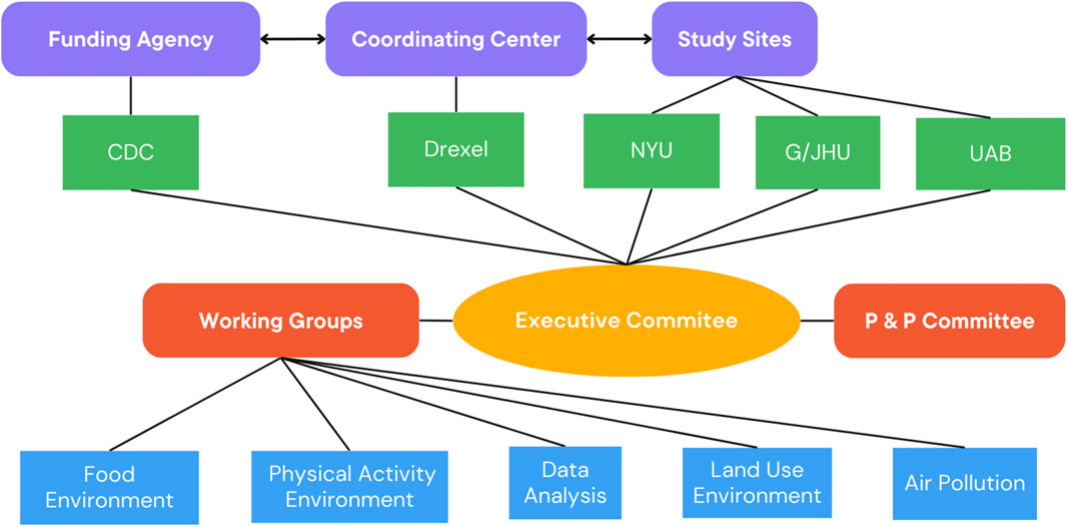
Organizational structure of the diabetes LEAD network.

Bringing together independent research teams across the USA into a cohesive and effective collaborative network involved substantial time and effort, from both administrative and scientific perspectives. Although many technological advances exist that facilitate research across locations, challenges still arise. First, the operational infrastructure of the LEAD Network (e.g., governance, decision-making body, working groups and network committees, types and frequency of meetings, and means of communications) had to be established (Figure 1). Second, establishing the collaborative research network necessitated administrative steps, including executing collaborative agreements and memoranda of understanding between participating institutions, securing institutional review board approvals at each institution, and establishing data use agreements when necessary. Based on our experience, we believe future networks would benefit from funding agencies providing clear guidance on the scope of the goals for collaborative agreements and take into consideration the time that a newly formed network takes to establish working relationships. These relevant administrative considerations of successful collaboration have been absent from existing literature on multisite research teams, perhaps because so few studies have focused specifically on epidemiological collaborations [9].

Members of the LEAD Network navigated interpersonal dynamics that come with new research partnerships, including variations in communication styles and approaches to research [10]. Effective communication was facilitated by holding annual in-person network-wide meetings, with time allocated to learning about each other’s strengths and expertise through formal scientific presentations at research meetings and in casual settings. The onset of the COVID-19 pandemic and resultant travel restrictions in the final years of our collaboration ended our network-wide in-person meetings; however, we were fortunate to have already forged strong relationships and gained familiarity with each other’s research approaches. It is difficult to know if we would have had the same success without the opportunity to develop relationships in person. Ongoing opportunities for in-person interaction and team building throughout the project period would likely have supported creative work and collaborative capacity, which is consistent with a body of evidence on the impact of team building on team productivity [4,7,11,12].

### Data access and data sharing

One challenge that has been largely unaddressed in previous literature is the variation in data management approaches across research teams and institutions, with different approaches to storing, analyzing, and sharing data (e.g., servers and software). Key data products were created centrally to share across the LEAD Network (as well as publicly); accounting for and accommodating varying data needs and preferences required an investment of time and troubleshooting creativity that went well beyond our initial expectations. For example, some institutional SPAM filters were more sensitive than others, and some firewalls did not allow certain types of file sharing. Further, working with sensitive data across multiple research sites and institutions was logistically challenging. Each site used data that contained protected health information and thus were not able to easily share those data across the network.

To address these barriers, we adopted a flexible federated model opting to keep data housed and secured at each respective research site rather than transfer sensitive data to the Lead Network’s Coordinating Center for analyses. This required additional coordination of data management and analyses across the network but ultimately saved time that would have been required to put into place a more comprehensive set of data use agreements. Sites took a more active role in advancing both network-wide analyses and site-specific analyses, and the LEAD Network Coordinating Center prioritized developing new data products (e.g., measures of environmental and community exposures related to T2D), guiding analytic decisions, and reviewing code. We found success by setting up a formal process for code sharing and review through our data analysis working group, using Microsoft® Office 365® for sharing documents and data and using SmartSheet® for tracking purposes. Overall, flexibility to work with different technology, tools, and platforms proved useful to guide our collaborations.

We recommend having working groups to facilitate discussion around how to balance network goals with each institution’s and research team’s preferred practices regarding communication tools, file and data sharing, processing, analyses, and documentation. Working groups are most effective when they include each site’s data manager, analytic leads, and legal staff and could mitigate data challenges by building in, up-front, processes for code sharing, review, and validation across sites to increase consistency and transparency. Time and funding need to support personnel in maintaining this aspect of the network, from data access and acquisition to analysis and attempts at reproducing results across sites [13]. Planning to establish a governance infrastructure allows investigators to focus on scientific aims of the network. Opportunities also exist for funding agencies to ensure these efforts can occur smoothly by prespecifying data sharing requirements as part of the RFA and allocating time and funding to support these time-intensive efforts.

### Aligning network research aims

The first objective of the LEAD Network was to develop a consistent set of network-wide aims. Although each research site had *a priori* identified specific aims for their site-specific work, the network structure necessitated new, network-wide scientific aims that could be achieved by harmonizing approaches across the three research sites and study populations. Coming to consensus on network-wide specific aims was a lengthy process. We wished to maximize scientific impact of the network through the coordinated and thoughtful use of different study population data that covered different geographies and had different origins (i.e., EHRs vs. survey-based longitudinal cohort data), a consideration that is largely unique to epidemiologic multi-site studies. We had to collectively learn about each study population and dataset, including its strengths and weaknesses, and strategize how to navigate data and inferential limitations while harmonizing analytic approaches across three datasets. Investigators at all study sites engaged in a dialogue to come to consensus on what gaps in knowledge could be answered with our disparate datasets and how our collective efforts could be greater than the sum of their parts.

The LEAD Network held multiple meetings, each with a prespecified objective, which helped ensure accountability in research progress. To prioritize inclusiveness and transparency, requests for input varied in format with opportunities for all voices of the network to be heard. Meetings included online and in-person modalities, with large and small groups of varying composition; input was also sought in writing. The LEAD Network Coordinating Center documented decisions in meeting minutes and created a network decision log to keep track of important discussions and decisions related to the scientific aims of the network. Throughout, we were able to build consensus on conceptual frameworks of how we believed neighborhood exposures may impact the geographic distribution of T2D and collectively agree on achievable and important scientific aims for the network, with attention to feasibility of research timelines.

### Harmonization of measures and methods

To address our network’s research aims, we needed to: 1) define T2D outcomes across the study sites; 2) choose important relevant covariates, often assessed slightly differently across sites (e.g., smoking status and individual-level socioeconomic status); and 3) achieve consensus on analytic approaches (i.e., model specification, time periods of analyses, and approach for exposure assignment to individuals). To harmonize analyses in ways that would yield scientifically meaningful evidence, we opted to define a set of coordinated exposure variables and metrics, derived and assembled at our coordinating center. For each exposure variable, a site with relevant expertise led the collaborative process to finalize measurement, partnering with the coordinating center and experts across sites to generate the metrics. This information was shared with all network members, with consideration of alternatives and refinements from the full range of members’ expertise. Working groups played a leading role in integrating feedback, informing the development of data sources now available through the LEAD Network and available for download via the University of Michigan’s Institute for Social Research (ICPSR): https://doi.org/10.3886/ICPSR38645.v1.

Across the LEAD Network, we had wide variation in the individual-level health datasets, ranging from a national sample including all Veteran’s Affairs (VA) EHR data (*n* = 6,082,246), to the national REGARDS cohort (*n* = 11,208), to EHR data from a health system primarily serving rural Pennsylvania (Geisinger, *n* = 578,485) [8,14]. Given the broad geographic extent of our network’s population health data coverage, this effectively meant that we needed to develop measures that spanned the US, were applicable to the local environment in scale, and were applicable to communities ranging from very rural to very urban. While the large geographic extent of our study populations was an asset, it also hindered stakeholder and community input due to the diversity in the communities represented by our network’s study areas. The same geographic scale (e.g., county level, census tract level, and block level) was not necessarily relevant across all three cohorts due to differences in levels of urbanicity; however, to harmonize our analyses, it was necessary to use the same scale.

To facilitate harmonization of analyses while accounting appropriately for differences in study design, a Data Analysis Working Group was formed and included statisticians and epidemiologists from all study sites. The group collaboratively drafted a statistical analysis plan (SAP) for each proposed manuscript, outlining the approaches each site would take to answer the same questions with their specific data and updating this documentation regularly as decisions were made. The SAP had to be specific enough to ensure that each site could answer the questions we posed jointly, but general enough to account for site differences, such in variable specifications, study designs (and thus different underlying models), and geographies. While many collaborative studies and research networks have data analysis components, having flexible SAPs is especially important for the unique and population-specific approaches needed for conducting multi-cohort collaborative epidemiology studies. Variation in measures and outcomes has been observed in previous epidemiology multisite collaborations. Previous studies, however, have not provided plans for addressing these issues in future work[15]. These challenges ultimately provided our study teams opportunities to learn new methods and consider how to answer the same questions in multiple ways. While more complex than analyzing pooling data across multiple similarly designed studies, this collaborative learning process and the resulting papers can help inform efforts to improve reproducibility and external validation in the public health literature.

Budgeting time to create unified research protocols across sites early in the research process improves the ability to harmonize metrics and adapt methods to diverse study designs. Exploring how to measure constructs, sometimes with different variables across sites, can resolve differences in jargon across experts from diverse backgrounds and training. Study leadership should direct these conversations to closure, noting the strengths and limitations of decided approaches. Networks conducting these tasks should approve a process for making scientifically justified decisions about measures and constructs after weighing the strengths and limitations identified by the entire research team, and this can be facilitated by the coordinating center [16].

Early efforts to describe each study population can help researchers understand new datasets and help inform analytic approaches that are sensitive to study population unique characteristics. We recommend that large collaborative research groups have conversations early and often about addressing reproducibility and external validity, which are central to learning from a collaborative research effort. Similarly, if newer or non-standard methods are required to answer network research questions, we recommend that educational and training opportunities specific to these methods be included in the network plan for trainees, statisticians, data managers, and other members of the research team to be well-versed in the required approach. Overall, being realistic about the substantial time and resources it takes to harmonize variables and analytic approaches for multisite research efforts is essential to ensure a smooth process for harmonizing measures and adapting methods to studies with differing designs, although this is often at odds with timelines from funding agencies.

### Dissemination of findings

Because many individuals were involved in the LEAD Network, we needed to clearly define authorship roles. Each site included several researchers, spanning career stages and contributing in various ways. We aimed to determine authorship fairly while also promoting junior researchers and trainees. Ultimately, many of our network-wide papers included so many co-authors that the drafting process became unwieldy. To alleviate this issue, we created small writing groups to draft manuscripts once all co-authors had approved the proposal and then employed the larger group of co-authors to edit and revise tables, figures, and manuscript drafts. The formalization of a publications and presentations (P&P) committee (described in more detail below) helped to ensure equity in authorship and that co-author lists included members from each site. This challenged us to consider how to reward the work that was done, how to “distribute” credit across senior scientists leading efforts at different sites, and how to navigate the timelines of getting papers to press when there are many co-authors. While addressing these challenges may have extended our timelines, we believe obtaining input from a variety of experts enhanced the quality of our work.

We established the P&P Committee with representatives from each site to review research proposals and manuscripts for network-wide and site-specific analytic work and track our research output, documenting achievement of our scientific aims. This improved the quality of research products, providing multiple opportunities for peer review *prior* to submission of research findings to conferences and journals. The process was especially helpful for junior investigators and trainees involved in the LEAD Network, giving them the opportunity to interact with and receive feedback from senior researchers. This also limited unintentional redundant efforts and ensured consistency in the descriptions of the LEAD Network and data products across manuscripts and ensured accountability in the completion of proposed manuscripts through the publication process [11]. As of September 2024, LEAD Network investigators have published 30 related manuscripts and presented findings at over 18 research meetings, with more in the pipeline.

## Discussion

**Figure 2.**
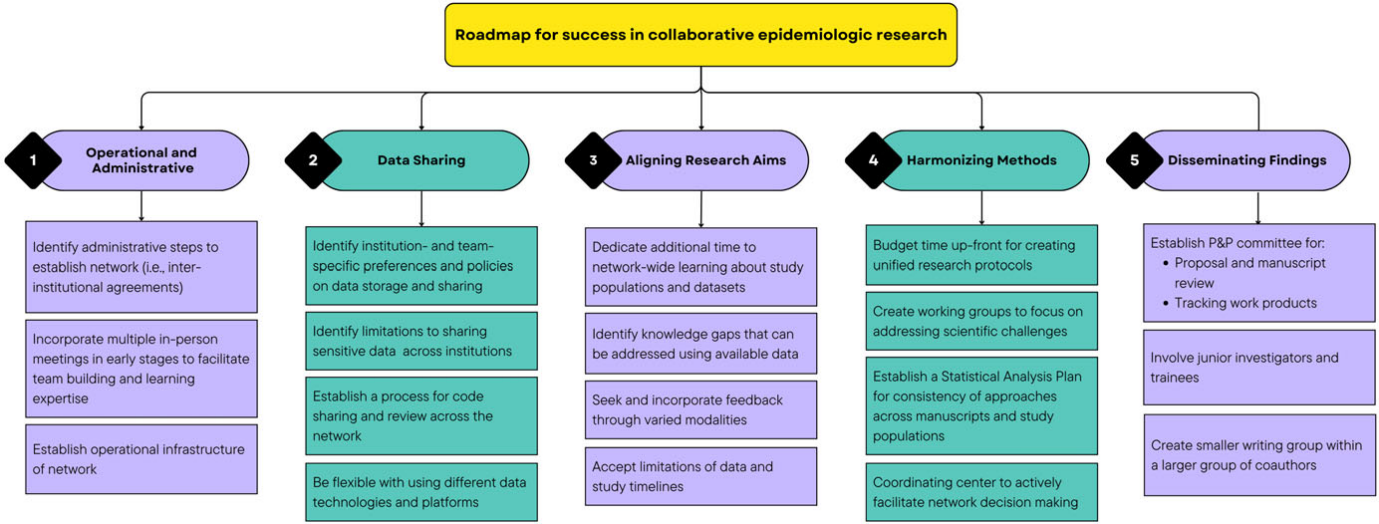
Roadmap for success in collaborative epidemiologic research.

We summarize our recommendations in a roadmap for success (Figure 2), which future collaborative networks in epidemiology can use as a checklist to facilitate success in several functions across the lifespan of the project. These recommendations reflect areas in which we found specific guidance in prior studies to be lacking, particularly for collaborations of epidemiologic studies. The categories of operational and administrative processes; data access and sharing; aligning research aims; harmonizing methods; and disseminating findings broadly cover the kinds of challenges unique to multisite and multi-organizational networks conducting epidemiologic research. We found that guidance in these categories was previously lacking in prior literature on multisite research collaborations. Broadly, we emphasize that early planning and support for administrative and technological infrastructure can help mitigate challenges associated with conducting research across institutions and geographies, which could be facilitated through infrastructure grants from funding agencies. We emphasize that future networks should account for tasks taking a longer time than anticipated since bringing together disparate groups incurs greater challenges than bringing together researchers with already established research relationships and infrastructure. Nonetheless, collaborative research networks have enormous potential for scientific innovation and advancement; by anticipating the operational challenges, we hope that future networks can focus more time and energy toward scientific goals and contributions, thereby answering important scientific questions more effectively and efficiently.

In our experience with the LEAD Network, we found that achieving consensus was often difficult, and future research networks should consider how to best facilitate decision-making. We recommend implementing a defined process (standard operating procedure) to speed up decision-making related to study measures and approaches, as often there are many ways to measure or tackle scientific questions, and often no sole correct approach. To complement this, we also recommend that funding agencies that administer and oversee cooperative agreements should identify leadership from the funding agency who can help guide the work so that it is consistent with the agency’s goals and specify the expected timeframes for their input at key decision points and during manuscript development. Coordinating centers can lead consensus building and help to establish clear processes for decision-making.

Collaborative research is difficult but worthwhile. There is growing evidence that team science and collaboration can produce innovation; empirical work has demonstrated that collaborative work and coordinating centers, specifically, can advance research translation for certain diseases (e.g., cancer) [16]. Further, such research networks offer opportunities for active involvement by junior researchers and trainees at the multiple stages of the research process, as has been noted by other similar network-based studies [17]. It is worth noting that, apart from measuring publication output, evaluating markers of success of such networks is challenging in the short-term. Previous evaluation of collaborative team science has noted that often the benefits of such efforts are realized years after a network’s conception [5]. To ensure the benefits of collaborative team science are realized, funding agencies could commit resources to support networks’ longevity. Reflecting on our experiences with the Diabetes LEAD Network, we believe that we were successful in our efforts and articulate that a proactive approach to anticipating and addressing challenges of conducting collaborative research can maximize opportunities and strengths of multisite research networks in epidemiology.
